# The flatness of bifurcations in 3D neuronal branching patterns

**DOI:** 10.1186/1471-2202-12-S1-P349

**Published:** 2011-07-18

**Authors:** Jaap van Pelt, Harry BM Uylings

**Affiliations:** 1 Computational Neuroscience Group, Department of Integrative Neurophysiology, CNCR, VU University Amsterdam, De Boelelaan 1085, 1081 HV Amsterdam, The Netherlands; 2 Dept. Anatomy & Neuroscience, VU University Medical Center, P.O. Box 7057, 1007 MB Amsterdam, The Netherlands

## 

The geometry of bifurcations in natural branching systems often reflects optimization constraints during formation, leading to, for instance, a planar arrangement of the branches (segments). The present study aimed at testing whether bifurcations in 3D neuronal branching patterns also follow this general property. A measure for the flatness of bifurcations is the apex angle of the right circular cone enwrapping the bifurcation (cone angle) and earlier applied to dendritic bifurcations [1]. Recently, the cone angle was used by Kim et al. [2] in an analysis of bifurcations in Purkinje cells and retinal ganglion cells. As the cone angle distributions in these cells resembled those of random bifurcations it was concluded that the observed flatness already naturally emerges from random conditions.

**Figure 1:**
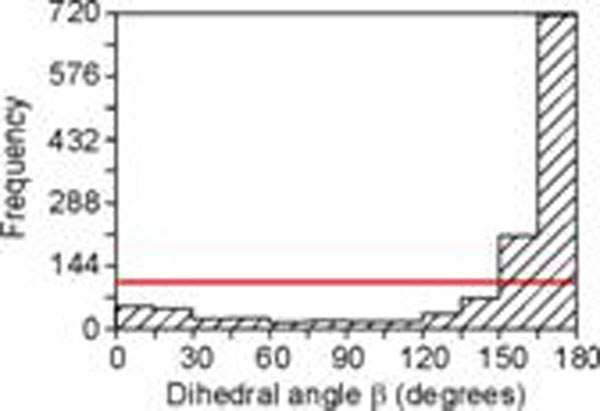
Frequency distribution of the dihedral angle β of random bifurcations (horizontal line), and of basal dendritic bifurcations (dashed histogram).

In the present study, a number of different geometrical measures of flatness has been developed and evaluated on their power for expressing the flatness of 3D bifurcations. These measures are applied to bifurcations from rat visual cortex pyramidal basal dendrites, and to random bifurcations (i.e., bifurcations with random oriented segments in 3D space). Frequency distributions of the various flatness measures of random and dendritic bifurcations appeared to be significantly different (Kolmogorov-Smirnov test). Dendritic bifurcations appeared to be more planar than random ones. In addition, parent segments in dendritic bifurcations have a strong preference to be aligned oppositely to the angular bisector of the daughter segments. These findings suggest that dendritic bifurcations are also formed under the influence of optimality constraints during the dendritic branching process. The flatness measure most strongly expressing the flatness of a 3D bifurcation was found to be the dihedral angle β between the daughters’ plane and the plane formed by the parent segment and the line in the daughters’ plane perpendicular to the angular bisector of the daughter segments. The dihedral angle β has a uniform distribution for random bifurcations but is highly skewed for dendritic ones (Fig. 1).

We conclude that 3D bifurcations in rat cortical pyramidal basal dendrites are significantly more flat than random bifurcations. In addition, parent segments are preferentially aligned opposite to the angular bisector of the daughter segments. These findings are in line with findings in tissue culture that neuritic bifurcations are subjected to elastic tensions [3], minimizing the distances between the nodes comprising the bifurcation.
